# Correction: Ethnic differences in maternal diet in pregnancy and infant eczema

**DOI:** 10.1371/journal.pone.0246481

**Published:** 2021-01-28

**Authors:** The PLOS ONE Staff

## Notice of Republication

This article was republished on May 22, 2020, to correct errors in the byline that were introduced during the typesetting process. The publisher apologizes for the errors. Please download this article again to view the correct version. The originally published, uncorrected article and the republished, corrected articles are provided here for reference.

## Supporting information

S1 File Originally published, uncorrected article. (PDF)

S2 File Republished, corrected article. (PDF)
