# Supplementary material for: Ethnic differences in maternal diet in pregnancy and infant eczema
Source: PLoS One. 2020 May 14;15(5):e0232170. doi: 10.1371/journal.pone.0232170 (PMC7224524; doi:10.1371/journal.pone.0232170)
Supplement: S1 Table — Reprinted with permission39. (PDF) [file pone.0232170.s001.pdf]

Table S1: Food items with a loading score ≥ |0.30| that characterize each of the three dietary patterns. Reprinted with permission[39].

Food Group	Plant-based	Western	Balanced
Fats		0.54	
Full Fat Dairy			
Low Fat Dairy	0.40	0.40	
Fermented Dairy	0.61		
Meat	(-0.36)	0.41	0.32
Eggs			0.37
Organ Meats			
Fish and Seafood			0.50
Processed Meats		0.55	
Meat Dishes			0.49
Poultry and Waterfowl			0.36
Fried Foods			
Leafy Greens			0.38
Cruciferous Vegetables			0.55
Legumes	0.62		
Fresh Seasonings	0.72		
Starchy Vegetables		0.42	
Vegetable Medley	0.42		0.48
Other Vegetables	0.70		0.32
Tofu			
Fruits			0.53
Whole Grains	0.71		
Refined Grains			0.35
Pasta		0.55	
Pizza		0.32	
French Fries		0.47	
Non-Meat Dishes	0.63		
Stir-Fried Dishes			0.47
Snacks		0.43	
Nuts and Seeds			0.34
Sweets		0.47	
Condiments		0.48	0.41
Tea	0.56		
Coffee		0.33	
Sweet Drinks		0.55	
Artificial Sweets			
Eigenvalue	4.08	3.14	3.05
Cumulative Variation	0.11	0.20	0.29
