# Supplementary material for: Ethnic differences in maternal diet in pregnancy and infant eczema
Source: PLoS One. 2020 May 14;15(5):e0232170. doi: 10.1371/journal.pone.0232170 (PMC7224524; doi:10.1371/journal.pone.0232170)
Supplement: S2 Table — The exact questions as presented on questionnaire completed by mother for each of the cohorts, and a harmonized definition for each covariate. (PDF) [file pone.0232170.s002.pdf]

Table S2: Harmonized definitions. The exact questions as presented on questionnaire completed by mother for each of the cohorts, and a harmonized definition for each covariate.

Outcome	CHILD	FAMILY	START
Infant Eczema	Has your child had a rash in the last 6 months? If seen by a healthcare professional, what was the diagnosis of this rash (Options include eczema/atopic dermatitis)	Has your child ever been diagnosed with eczema?	Has your child been diagnosed with eczema by a doctor?
Harmonised definition: Has your child been diagnosed with eczema/atopic dermatitis?			
Maternal eczema	Have you ever had skin allergy symptoms (eczema, hives, allergic rash)?	Have you ever been diagnosed by a doctor as having eczema, prior to this pregnancy?	Have you ever been diagnosed, by a doctor, with eczema (not during pregnancy)?
Harmonized definition: Have you ever had skin allergy (including eczema)?			
Breastfeeding at 1-year	Is infant currently being breastfed; when did breastfeeding stop	Was infant breastfed, baby's age when breastfeeding stopped	Was infant breastfed, baby's age when breastfeeding stopped
Harmonized definition: Infant is still being breastfed (exclusive or non-exclusive) at 1-year visit.			
Ethnicity	Mother's ethnic group: white European, Indo-Canadian, Black, Japanese, Filipino, Middle Eastern, Chinese, Southeast Asian, South Asian, First Nations, Hispanic	Mother's ethnic group: Hispanic, European, South Asian, Arab, East/Southeast Asian, African, Indigenous	all South Asian
Harmonized definition: Ethnic groups included: white European, South Asian, East/South East Asian, Indigenous/Indigenous, African, and Other.			
