# Supplementary material for: Correction: Ethnic differences in maternal diet in pregnancy and infant eczema
Source: PLoS One. 2021 Jan 28;16(1):e0246481. doi: 10.1371/journal.pone.0246481 (PMC7842987; doi:10.1371/journal.pone.0246481)
Supplement: S1 File — (PDF) [file pone.0246481.s001.pdf]

PLOS ONE

RESEARCH ARTICLE

Ethnic differences in maternal diet in pregnancy and infant eczema

Michael A. Zulyniak[1,2], Russell J. de Souza[3,4], Mateen Shaikh[3], Chinthanie Ramasundarahettige[4], Keith Tam[1], Natalie William[1], Dipika Desai[4], Diana L. Lefebvre[1], Milan Gupta[1,5], Padmaja Subbarao[6,7], Allan B. Becker[8], Piushkumar J. Mandhane[9], Stuart E. Turvey[10], Theo Moraes[6], Meghan B. Azad[11], Koon K. Teo[1,4], Malcolm R. Sears[1,3,4]*, on behalf of the *NutriGen* Alliance investigators¶

1 Department of Medicine, McMaster University, Hamilton, Ontario, Canada, **2** School of Food Science and Nutrition, University of Leeds, Leeds, United Kingdom, **3** Department of Health Research Methods, Evidence, and Impact, McMaster University, Hamilton, Ontario, Canada, **4** Population Health Research Institute, Hamilton Health Sciences and McMaster University, Hamilton, Ontario, Canada, **5** Canadian Collaborative Research Network, Brampton, Ontario, Canada, **6** Hospital for Sick Children & Department of Paediatrics, University of Toronto, Toronto, Ontario, Canada, **7** Department of Physiology, University of Toronto, Toronto, Ontario, Canada, **8** Department of Immunology, Faculty of Medicine, University of Manitoba, Winnipeg, Manitoba, Canada, **9** Department of Paediatrics, Faculty of Medicine and Dentistry, University of Alberta, Edmonton, Alberta, Canada, **10** Department of Paediatrics, Faculty of Medicine, BC Children's Hospital and Child and Family Research Institute, University of British Columbia, Vancouver, British Columbia, Canada, **11** Department of Pediatrics and Child Health, Health Sciences Centre, Children's Hospital, University of Manitoba, Winnipeg, Manitoba, Canada

¶ Membership of the Nutrigen Alliance is listed in the Acknowledgments.
* anands@mcmaster.ca

OPEN ACCESS

Citation: Zulyniak MA, de Souza RJ, Shaikh M, Ramasundarahettige C, Tam K, William N, et al. (2020) Ethnic differences in maternal diet in pregnancy and infant eczema. PLoS ONE 15(5): e0232170. https://doi.org/10.1371/journal.pone.0232170

Editor: Pal Bela Szecsi, Copenhagen University Hospital Holbæk, DENMARK

Received: October 8, 2019

Accepted: April 8, 2020

Published: May 14, 2020

Copyright: © 2020 Zulyniak et al. This is an open access article distributed under the terms of the Creative Commons Attribution License, which permits unrestricted use, distribution, and reproduction in any medium, provided the original author and source are credited.

Data Availability Statement: All relevant data are within the paper and its Supporting Information files. Raw data cannot be shared publicly because of lack of participant consent, but are available upon request to Kathy Stewart, McMaster University at: Email: kmstew@mcmaster.ca Phone: 905-525-9140 Ext. 21523.

Funding: This work was supported by funding by a Canadian Institute for Health Research (CIHR) Grant in Food & Health Population Health Research grant [RFA# 201301FH6; 2013-2018; S.A.A., A.B.

Abstract

Background

The global prevalence of childhood eczema has increased over the last few decades, with a marked increase in high-income countries. Differences in prevalence of childhood eczema between countries and ethnicities suggest that genetic and early modifiable environmental factors, such as dietary intake, may underlie this observation. To investigate the association between pregnancy diet and infant eczema in a consortium of prospective Canadian birth cohorts predominantly comprised of white Europeans and South Asians.

Methods

We evaluated the association of maternal dietary patterns reported during pregnancy (assessed at 24–28 weeks gestation using a semi-quantitiative food-frequency questionnaire) with parent-reported physician-diagnosed infant eczema at 1-year from 2,160 mother-infant pairs. Using three dietary patterns ("Western", "plant-based", and "Balanced") previously derived in this cohort using principal component analysis, we used multivariable logistic regression to determine the association of these dietary patterns with infant eczema, adjusted for potential confounders.

Results

We observed a lower odds of eczema in the full sample combining white Europeans and South Asians with greater adherence to a plant-based (OR = 0.65; 95% CI: 0.55, 0.76;

B., J.B., R.J.dS., M.G., M.R.S., P.S., G.W.] and a CIHR RCT Fellowship grant [MTP201410; M.A.Z.]. START data was collected as part of a bilateral ICMR /CIHR funded programme [INC-109205] and HSF Canada Grant in Aid [NA7283]. CIHR provided funds for CHILD [AEC85761; A.B.B, P.M., M.R.S., P.S., S.E.T.), FAMILY [K.K.T.], data collection, with additional funding from AllerGen NCE Inc. [S.S.A]. M.R.S holds the AstraZeneca Chair in Respiratory Epidemiology at McMaster University. S.S.A. holds a Canada Research Chair Tier 1 in Ethnic Diversity and Cardiovascular Disease and a Heart and Stroke Foundation / Michael G. De Groote Chair in Population Health Research. M.B.A. holds a Canada Research Chair in the Developmental Origins of Chronic Disease. All authors declare no conflict of interest and that all funders (including AllerGen NCE Inc and AstraZeneca) had no role in study design, data collection and analysis, decision to publish, or preparation of our manuscript. Therefore, there was no influence on our adherence to PLOS ONE policies on sharing data and materials."

Competing interests: All authors declare no conflict of interest and that all funders (including AllerGen NCE Inc and AstraZeneca) had no role in study design, data collection and analysis, decision to publish, or preparation of our manuscript. Therefore, there was no influence on our adherence to PLOS ONE policies on sharing data and materials."

<0.001) and Western dietary pattern (OR = 0.73; 95% CI: 0.60, 0.89; P<0.01), after adjusting for other known predictors of eczema, including ethnicity, which was not significant. No associations were observed for the balanced diet. An interaction between the Western diet and ethnicity was observed (P<0.001). Following stratification by ethnicity, a protective association between the plant-based diet and infant eczema was confirmed in both white Europeans (OR = 0.59; 95% CI: 0.47, 0.74; P<0.001) and South Asians (OR = 0.77; 95% CI: 0.61, 0.97; P = 0.025). In white Europeans only, a Western diet was associated with a lower odds of infant eczema (OR = 0.69; 95% CI: 0.56, 0.87; P = 0.001) while a balanced diet increased the odds of infant eczema (OR = 1.23; 95% CI: 1.02, 1.49; P = 0.03). Beyond a plant-based diet, no significant associations with other dietary patterns were observed in South Asians.

Conclusion

A plant-based diet during pregnancy is associated with a lowered odds of infant eczema at 1 year in all participants. Future studies of the components of plant-based diet which underlie the lower risk of eczema are needed.

Introduction

Atopic diseases, including eczema, are characterized by an immune response to normally innocuous antigens in the environment. [1] The global prevalence of atopic diseases in children has increased over the last few decades, with a marked increase in developed nations. [2, 3] In Canada, the prevalence of childhood atopic diseases such as eczema is 20–25% [4, 5]. In Europe, the Generation R study reported that, compared to predominantly white European Dutch children, children with parents of African, Caribbean, or South American origin have a higher risk of eczema before the age of four years. [6] However, in the United Kingdom, white Europeans have a higher prevalence of eczema (35.8%) than children of Pakistani (23.6%) or other non-white ethnic groups (25.8%), even after accounting for common risk factors, such as sex, birth weight, breastfeeding, family history, exposure to smoke, and socioeconomic status. [7] These differences in prevalence between countries and ethnicities point to a complex interplay between genetic and potentially modifiable environmental factors, including diet, in the development of eczema. [8, 9]

Diet during pregnancy is an early environmental exposure that has been associated with eczema. [10–14] However, dietary intake varies markedly between ethnic groups [15] and may partially account for inconsistencies between randomised controlled and prospective cohort studies on the association between diet and supplementation (e.g., fish oils, probiotics, and prenatal multivitamins) and allergic disease, including eczema. This, along with high degrees of heterogeneity of dietary assessment and reporting between studies limit the use and interpretability of meta-analyses.

A recent meta-analysis reported divergent results between 3 prospective cohort studies regarding the association between maternal vegetable intake during pregnancy and infant risk of eczema at 2 years of age. [11] Two studies (Japan, = 762; Norway, n = 3086) reported a pooled protective effect of maternal intake of vegetables during pregnancy (pooled OR = 0.71, 95% CI = 0.53–0.96), whereas the third study (Spain and Greece; n = 2516) reported a near significant increase in risk of eczema (RR = 1.21, 95% CI = 0.97–1.51). In their meta-analysis of

32 cohort studies, the authors reported that individual nutrients, food groups, and dietary patterns consumed by the mother during pregnancy were not consistently associated with eczema. [11]

The effect of a single nutrient on a health outcome is often difficult to identify with certainty because of the complex matrix of nutrients within the foods we eat and their modification by preparation and cooking methods. [16] Therefore, studies focusing on single nutrient associations can misrepresent the effects of the nutrients when consumed in the typical manner as part of foods and dishes in a typical diet. [11] An alternative approach to studying individual nutrients is to study the dietary patterns that are most common within a population [17]. In diverse populations, rather than using pre-defined diet patterns (e.g., *Mediterranean*), data-driven patterns can characterise "natural" diet patterns that are prominent within the entire population [17]. This permits the association between a variety of prominent dietary patterns within a population and outcomes. [18, 19] In this paper, we investigate the association between maternal dietary patterns during pregnancy and infant eczema in an ethnically-diverse Canadian prospective birth cohort consortium.

Materials and methods

Study population

The NutriGen Alliance has been previously described. [15] Briefly, it is an ethnically diverse consortium of four Canadian birth cohort studies investigating the contribution of nutritional, genetic, and epigenetic factors to the health of pregnant women and their children—(i) the Canadian Healthy Infant Longitudinal Development (CHILD) Study [20]; (ii) the Family Atherosclerosis Monitoring In earLY life (FAMILY) Study [21]; (iii) the SouTh Asian birth cohoRT (START) [22]; and the Aboriginal Birth Cohort (ABC) [23]. Ethical approval was obtained independently for all studies from the Hamilton Integrated Research Ethics Board—CHILD (REB 07–2929), FAMILY (REB 02–060), START (REB 10–640) and ABC (REB 12–152). As of August 2018, 5,018 women with singleton pregnancies have provided comprehensive clinical and dietary data. At 1-year follow up, 2,765 mother-infant pairs have completed a child-health questionnaire that reported on a diagnosis of eczema. To ensure adequate statistical power the two largest ethnic populations, 2,305 women who reported either white European (n = 1,460) or South Asian (n = 845) ethnicity were selected for this analysis. Among these, 145 participants were excluded because the mother reported an implausible diet ($<$500 or $\geq$6,500 kcal/day) or did not report on $\geq$ 10 food frequency questions, leaving 1,378 white European and 782 South Asian mother-baby pairs in the final analysis.

Assessment of diet, harmonization, and diet pattern analysis

The development and validation of the dietary assessment tools used in the NutriGen cohorts, and the dietary patterns have been previously described. [15, 22, 23] Briefly, dietary food intake information during pregnancy was collected from mothers in each cohort using a semi-quantitative food-frequency questionnaire (FFQ) between 24–28 weeks gestation. The CHILD cohort used the Fred Hutchinson Cancer Center tool. [24] The FAMILY and START cohorts used ethnic-specific semi-quantitative FFQs developed for the Study of Health and Risk in Ethnic Groups (SHARE) study. [25] Prior to performing principal component analysis (PCA), 36 common food groups were created to aggregate individual FFQ items from each study according to nutrient profile and food type. [18, 26–28] We performed PCA with an orthogonal 'varimax' rotation [29], an approach that identifies dietary patterns (i.e., foods commonly consumed together) that best explain the dietary variability within the cohort. [26, 30–32] The number of dietary patterns retained were determined by visual inspection of scree plots in

conjunction with eigenvalues, and principal component interpretability. [33, 34] The PCA of the combined cohorts identified 3 dietary patterns (descriptions are provided in Results) that collectively explained 29% of variation in diet that was observed in the cohort. We called these PCA diet patterns 'plant-based' (dairy, legumes, vegetables, whole grains, and an aversion to meats; adherence range: -2.6 to +4.9), 'Western' (fats, meats, processed foods, and starchy vegetables; adherence range: -3.9 to +5.0), and 'balanced' (diverse sources of animal proteins (especially fish), vegetables, fruits, nuts & seeds; adherence range: -2.6 to +7.1) (see S1 Table for greater detail). A PCA adherence score for each pattern was obtained for each mother. A higher score reflected greater intake of the foods that loaded positively on a dietary pattern (i.e., loading score > 0.30) and reduced consumtpion of foods that loaded negatively on a dietary pattern (i.e., loading score < -0.30). If a food did not not load strongly (i.e., loading score $< |0.30|$) for a particular dietary pattern, this reflected that the intake of this food did not differ between high and low consumers of the dietary pattern. The adherence scores were adjusted to the mean total population energy intake (2500 kcal per day) using the residual method. [35, 36]

Assessment of eczema

The primary outcome of this analysis was a physician-diagnosis of eczema among children at age 1 year as reported by the parent completing a child health questionnaire; this assessment was available in all 3 participating cohorts in this analysis (see S2 Table). Data were harmonized across the cohorts by creating common definitions for each outcome.

Measurement of other variables

Parity, breastfeeding, pre-pregnancy weight, smoking history, ethnicity, post-graduate education, marital status, employment status, total household income, and maternal and paternal atopic disease diagnoses were self-reported. Gestational diabetes status was determined by self-report by the mother (CHILD) and through medical records or using an oral-glucose tolerance test (FAMILY and START) using the International Association of the Diabetes and Pregnancy Study Groups (IADPSG) definition. [37] We obtained maternal age, height, and gestational age, season of birth, length, and weight of offspring at birth from participants' medical records. Last measured maternal pregnancy weight was obtained from medical records at time of birth (FAMILY and START) or using a combination of medical records and maternal recollection (CHILD).

Statistical analysis

We performed statistical analyses using R (v.3.3.2). We summarized the distribution of exposures and covariates as means (standard deviation) for continuous variables or counts (%) for categorical variables; we assessed between-groups differences using ANOVA (continuous variables) or chi-square test (categorical variables). The majority of maternal and infant data in the CHILD cohort were complete; however, final pregnancy weight was not reported for 43% of CHILD mothers. We imputed missing values for final pregnancy weight in this cohort using the *Amelia II* package (v.1.7.4). [38, 39] We identified known and suspected risk factors for childhood eczema *a priori* and assessed them for inclusion in our model using a three-step method: (i) we entered all variables into a simple linear regression with eczema; (ii) we entered variables with $\alpha \leq 0.10$ into a forward stepwise selection procedure with other prospective variables; (iii) we retained variables significant at $\alpha < 0.05$ in the stepwise multivariable model as covariates in the final model. The *a priori* covariates exposure to breastfeeding and gestational age at birth did not satisfy the cutoff but we forced them into the model based on prior

knowledge. We assessed the association between maternal diet (diet pattern or diet adherence score) and covariates on eczema in infants at 1-year with a multivariable logistic regression model. To determine if the effect of maternal diet on eczema differed by ethnic group, we added a multiplicative interaction term to the model (maternal diet*ethnicity). We had > 80% power for detecting associations between dietary patterns and eczema.

Results

Demographic and clinical parameters

Maternal demographic and clinical parameters for the white European (n = 1,378) and South Asian (n = 782) participants are presented in Table 1. There were some notable ethnic variations in maternal exposures. South Asian women had a much higher prevalence of gestational diabetes (15% vs 1%), were more likely to be vegetarian (37% vs 3%) and almost all had never smoked (99% vs 69%). White European women were much more likely to report a personal history of eczema (46% vs 6%) and to report owning a furry pet (60% vs 5%). We did not observe an association between season of birth and infant risk of eczema as reported by others [40], which may be due to the differences in climate and seasons between regions of recruitment in Canada—Vancouver, Winnipeg area, and Southern Ontario.

Dietary patterns

We identified three orthogonal dietary patterns based on a previous analysis [41] (S1 Table). The PCA assigns a continuous score to each participant which indicates their degree of adherence to each of the three scores. These scores can be positive (indicating adherence) or negative (indicating avoidance) for each participant and are independent of one another. Each of the three patterns was characterised by the foods which loaded greater than 0.30 or less than -0.30. The plant-based dietary pattern (range: −2.6 to +4.9) was characterized fruits and vegetables, whole grains, and avoidance of meats; the Western diet pattern (range: −3.9 to +5.0) was characterized by high intakes of processed meats and foods, starchy vegetables, and red meats; and the balanced diet (range: −2.6 to +7.1) included a diverse range of food groups, including meats, vegetables and fruit, fish, and plant sources of proteins (e.g. nuts, soy). Foods that did not load $\geq|0.30|$ for a given pattern did not vary between high and low consumers of that pattern—e.g., 'fruit' and 'leafy greens' were not robust markers of adherence to a plant-based dietary pattern because the weekly consumption of 'fruit' and 'leafy greens' differed very little between individuals who's did or dd not resemble a plant-based dietary pattern.

Eczema

We observed a significant difference in the proportion of cases of eczema reported at 1-year, being substantially higher in white European (35%) than in South Asian (18%) infants ($P<0.01$) presented in Table 1. There was no association between the length of time the mother had lived in Canada and the infant risk of eczema.

Dietary patterns and infant eczema

In a model including both white Europeans and South Asians, the odds of infant eczema was reduced among offspring for every unit increase in PCA diet score in a mother's adherence to a plant-based (OR = 0.65; 95% CI: 0.56, 0.75; $P<0.001$) or Western dietary pattern (OR = 0.73; 95% CI: 0.60, 0.89; $P<0.01$). These associations were observed over and above the other known predictors of infant eczema, including maternal eczema (the strongest predictor, with OR = 2.07; 95% CI: 1.64, 2.62; $P<0.001$). No associations were observed for the balanced diet.

Table 1. Demographics of pregnant mothers and offspring.

Variable	White European	South Asian
	N = 1378	N = 782
**MOTHER**		
Age (yrs)	32.4 (4.7)	30.5 (4.1)
Pre-Pregnancy BMI (kg/m2)	25.1 (5.8)	23.7 (4.4)
Gestational Weight Gain (kg)	15.3 (5.8)	14.4 (6.2)
Height (cm)	165.7 (6.3)	162.1 (6.4)
Gestational Diabetes	15 (1%)	111 (15%)
Hypertension during Pregnancy	47 (3%)	23 (3%)
Eczema	630 (46%)	48 (6%)
Multi-Vitamin During Pregnancy	1165 (85%)	563 (72%)
Vegetarian	39 (3%)	290 (37%)
Calories per day (SD)	2056 (691)	1806 (670)
Gestational age at Delivery (wks, SD)	39.5 (1.2)	39.4 (1.1)
Primiparous	707 (51%)	332 (42%)
Smoking Status		
Never	955 (69%)	774 (99%)
Quit Pre-pregnancy	302 (22%)	2 (0%)
Quit during Pregnancy	62 (5%)	5 (1%)
Currently Smoking	58 (4%)	0 (0%)
Social Disadvantage index (SDI, SD)	0.4 (0.7)	1.1 (0.7)
Years in Canada (SD)	29.4 (8.1)	9.1 (8.8)
**INFANT**		
Birthweight (kg, SD)	3.5 (0.5)	3.3 (0.4)
Birth Length (cm, SD)	51.2 (2.4)	51.3 (2.6)
Ponderal Index (kg/m^3, SD)	26 (3.3)	24.2 (3.3)
Sex (Female)	612 (45%)	395 (51%)
Weight at 1 yr (kg, SD)	10.0 (1.3)	10.3 (1.6)
Ever Breastfed	1306 (95%)	769 (98%)
Breastfed at 1 yr	550 (40%)	338 (43%)
Physician-diagnosed Eczema	476 (35%)	137 (18%)
Baby Smoke Exposure		
None	1304 (95%)	492 (98%)
Minimal	50 (4%)	5 (1%)
Regular	22 (2%)	5 (1%)
**HOUSEHOLD**		
Number of Adults in home		
1	69 (5%)	6 (1%)
2	1150 (84%)	212 (48%)
≥3	155 (11%)	228 (51%)
Any furry Pet in House	823 (60%)	21 (5%)
Dog in House	563 (41%)	18 (4%)
Cat in House	425 (31%)	7 (2%)

https://doi.org/10.1371/journal.pone.0232170.t001

We then tested for an interaction between ethnicity and dietary patterns and observed a significant interaction for Western diet (P<0.001; Table 2). Following stratification by ethnicity (Table 3), a protective association between the plant-based diet and infant eczema was confirmed in both white Europeans (OR = 0.59; 95% CI: 0.47, 0.74; P<0.001) and South Asians

Table 2. Multiple variable regression models of diet patterns and infant eczema (n = 613 cases of eczema in 2160 children).

Infant Eczema	Odds Ratio (95% CI)	P value
Maternal Eczema (vs No)	1.93 (1.53, 2.45)	<0.001
Gestational Age (per wk)	1.01 (0.92, 1.1)	0.89
Ever Breastfed (vs Never)	0.74 (0.46, 1.21)	0.23
South Asian (vs White Europeans)	1.33 (0.9, 1.96)	0.15
**Plant-Based Diet**	**0.65 (0.55, 0.76)**	**<0.001**
Western Diet	0.87 (0.7, 1.07)	0.18
Balanced Diet	1.09 (0.92, 1.28)	0.32
Plant-Based Diet*South Asian		0.16
**Western Diet*South Asian**		**<0.001**

Overall $r^2 = 0.15$

Covariates were determined based on forward-step wise regression and entered into the model if p<0.05.

https://doi.org/10.1371/journal.pone.0232170.t002

(OR = 0.77; 95% CI: 0.61, 0.97; P = 0.025). In white Europeans only, a Western diet was associated with a lower odds of infant eczema (OR = 0.69; 95% CI: 0.56, 0.87; P = 0.001) while a balanced diet increased the odds of infant eczema (OR = 1.23; 95% CI: 1.02, 1.49; P = 0.03). Beyond a plant-based diet, no significant associations with other diet patterns were observed in South Asians.

Among South Asians, years lived in Canada was negatively associated with adherence to the plant-based diet pattern ($\beta = -0.039$; $r^2 = 0.12$; $P<0.001$) and positively associated with adherence to the Western diet pattern ($\beta = 0.013$; $r^2 = 0.05$; $P<0.001$) (Fig 1). A positive association was also observed for the balanced diet but much lower in effect size and degree of variance explained ($\beta<0.001$; $r^2 = 0.01$; $P<0.01$).

Discussion

In this multi-ethnic birth cohort consortium, we demonstrated that after considering known determinants of eczema, the patterns of maternal diet in pregnancy are associated with infant eczema at age 1 year. We found that the maternal plant-based diet was associated with a lower risk of infant eczema at 1 year in both ethnic groups. There was ethnic variation in the response to a maternal Western dietary pattern which is associated with lower risk of infant

Table 3. Multiple variable regression models of diet patterns and infant eczema stratified by ethnicity.

Infant Eczema	White Europeans n = 1378 (476 cases)		South Asians n = 782 (137 cases)	
	Odds Ratio (95% CI)	p-value	Odds Ratio (95% CI)	p-value
Maternal Eczema (vs No)	1.63 (1.27, 2.1)	<0.001	6.12 (3.03, 12.37)	<0.001
Gestational Age (per wk)	0.96 (0.87, 1.06)	0.40	1.17 (0.97, 1.41)	0.09
Ever Breastfed (vs Never)	0.83 (0.49, 1.4)	0.49	0.38 (0.11, 1.27)	0.11
Plant-Based Diet	**0.59 (0.47, 0.74)**	**<0.001**	**0.77 (0.61, 0.97)**	**0.025**
Western Diet	**0.69 (0.56, 0.87)**	**0.001**	1.03 (0.64, 1.65)	0.92
Balanced Diet	**1.23 (1.02, 1.49)**	**0.03**	0.72 (0.5, 1.05)	0.09

Covariates were carried forward from the full model, which was based on a forward-step wise regression and entered into the model if p<0.05.

https://doi.org/10.1371/journal.pone.0232170.t003

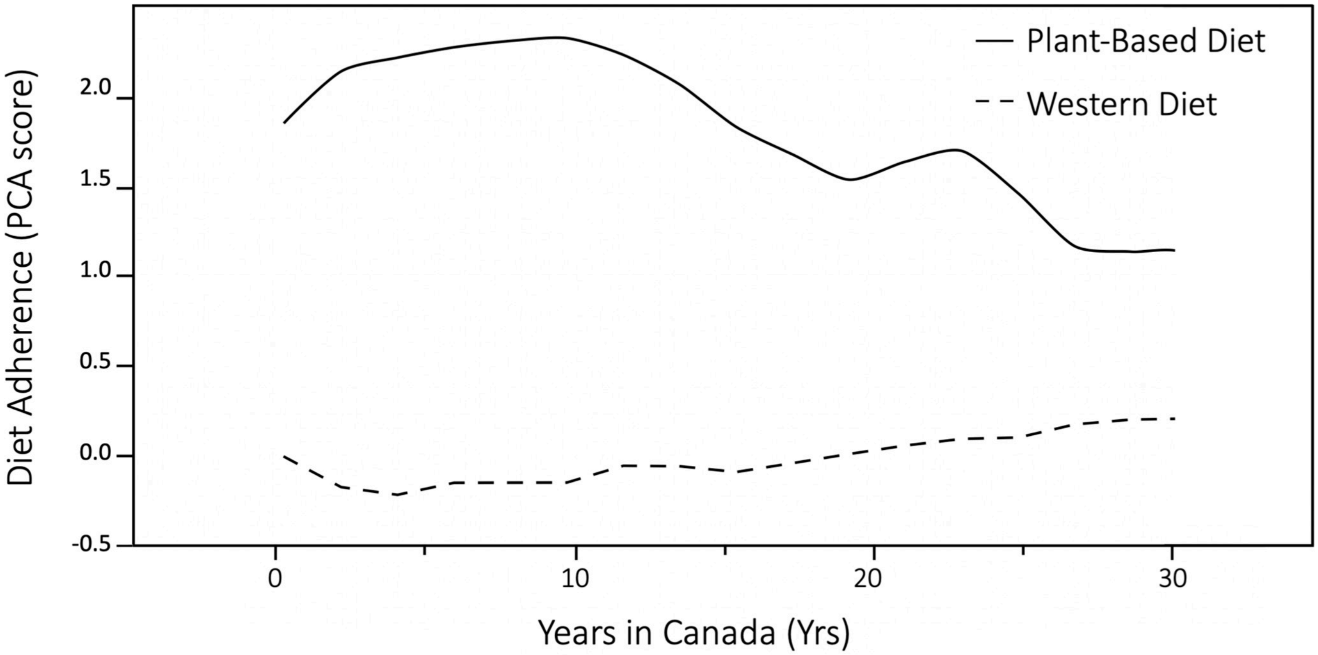

Fig 1. Association between years in Canada and PCA adherence scores to plant-based and Western diet patterns for South Asian participants.

https://doi.org/10.1371/journal.pone.0232170.g001

eczema, and the balanced diet which is associated with an increased risk of infant eczema among white Europeans, but not South Asians.

The plant-based diet is characterized by increased consumption of vegetable, legumes, whole grains, fermented and low fat dairy, and other non-meat dishes. Our observation that maternal consumption of plant-based diet is protective against infants developing eczema at age 1 in two ethnic groups is consistent with previous reports of a protective effect of green and yellow vegetables and dairy against eczema. [42] Previous prospective cohort studies in Japan (n = 763), [43] and Spain and Greece (n = 4,290) [44] did not observe associations between maternal dietary patterns and early infant eczema. This may be partly explained by their lower overall prevalence of eczema (19% in Japan [43] vs 28% in our study) and the fewer food groups captured on their Mediterranean diet score, [44] as compared to the comprehensiveness of our dietary patterns, which represent 36 high-order food groups. Neither a western nor balanced dietary pattern has been consistently associated with risk of infant eczema [10]. This inconsistency may arise from differences between study populations, and methods of data collection and analysis. The dietary patterns that we derived using PCA are comparable to diets commonly reported in literature; however, they also contain food items that do not fit all definitions—e.g., despite high weightings for plant-based foods, our 'plant-based' and 'Western' diets both contain 'low-fat dairy'. Interestingly, a recent meta-analysis[10] identified specific foods and micronutrients common to different diets which are associated with a reduced (e.g., probiotics and Vitamin D) [10, 42] or increased risk (e.g., high-sugar and meat consumption [45]) of eczema. Collectively, this may explain some of the unexpected associations that we observed, such as the Western diet's protective association with infant eczema, because dairy in Canada is fortified with Vitamin D. Future studies with greater ability to characterise nutrient intake and bioavailability with greater clarity—e.g., metabolite data—may be better suited to understand how subtle changes in maternal diet and nutrition may affect infant health.

South Asian women are more likely to consume a plant-based diet, explained in part by traditional dietary and faith practices; however, after immigrating to Canada, we and others [46] have observed that adherence to the Western diet increases over time and replaces more traditional plant-based diet foods (i.e., "dietary transition"). Such a transition may be gradual and correlate with other biological (i.e. gut microbiome) and cultural transitions that were not measured or accounted for in our analysis, but which may contribute to this change in risk and the observed difference with white Europeans.

The differing frequency of infant eczema between ethnic groups is consistent with a prior UK study which reported a lower incidence of eczema (20%) in South Asian children compared to white European children (32%). [7] We also report that South Asian women in our cohort were less likely to report a diagnosis of eczema than white European women. We did not identify previous reports of prevalence of adult eczema stratified by ethnicity, particularly for South Asians; however, evidence from the Born in Bradford birth cohort suggests that South Asian mothers are less likely to be diagnosed with either atopy or asthma than white British mothers (29% vs 58%). [7] The etiology for the observed disparity between ethnic groups is uncertain. [47] Interestingly, one small study (n = 25) recently demonstrated that patients with darker-skin perceive their symptoms of eczema as less severe compared to light-skinned patients, despite presenting comparably objective clinical signs of severity [48], suggesting that individuals of minority groups with darker skin (including South Asians) are more likely to be undiagnosed (i.e., false-negative). Newer diagnostic tools, such as the Eczema Area and Severity Index (EASI) that are exclusively objective and clinical may be better suited to diagnosing eczema in non-white ethnic groups.

Future studies should consider metabolomic or gut microbiome investigations to elucidate the biological pathways underlying our observation that maternal dietary intake is associated with the infant risk of eczema. Previously such lines of investigation have identified mechanisms by which preservatives common in foods and soft drinks (i.e., namely, sodium benzoate) increase the risk of infant eczema. [49] Such methodologies also lend themselves to Mendelian randomization studies if suitable genetic variants and biomarkers are available which could reduce confounding associated with dietary questionnaires and observational studies and infer causation with greater confidence. [50] Collectively, these approaches would provide strong evidence for a causal mechanism of plant-based diet on infant health and inform the design of randomized dietary intervention trials. Future studies, with data available, should also consider air pollution and climate during pregnancy as a mediator of risk for infant eczema. [51]

Our study has several strengths, including the inclusion of over 2000 women and infant pairs representing white Europeans and South Asians, use of a harmonized definition of eczema across all 3 cohorts, and the use of dietary pattern analysis. There are some limitations to our analysis, first the recruitment strategies varied by cohort (i.e. CHILD recruited from 4 sites across Canada; FAMILY from the Hamilton area in Ontario; START specifically recruited South Asians living in Peel Region in Ontario), and, hence the differential frequency of maternal and infant eczema by ethnicity may not be representative of the population. Secondly, our harmonised definition for maternal eczema (*'Have you ever had skin allergy*?') may be more readily recognised in white Europeans and add to higher prevalence in lighter skinned mothers. However, these do not affect the internal validity of our exposure (diet)–outcome (eczema) associations. Thirdly, we did not adjust for infant diet at 1-year of age because maternal and infant dietary patterns at 1-year are strongly correlated across major food groups (e.g., fruit, r = 0.54, P<0.001; vegetables, r = 0.42, P<0.001; snacks, r = 0.37, P<0.001) [52]. Finally, a standard commonly used definition (a parental report of physician-diagnosed eczema) was used for clinical assessment of eczema but residual confounding by study or study centre remains a

possibility and is a limitation for any multi-centre study with significant differences between study sites—e.g., ethnic diversity, socioeconomic status, and climate.

Conclusion

Maternal diet during pregnancy is associated with risk of infant eczema at 1 year. A plant-based dietary pattern is associated with reduced infant eczema in participants across multiple ethnicities. This could be considered by guideline developers and policymakers swhen developing dietary guidelines for pregnant women with respect to eczema. Future studies of the components of plant-based diet which may underlie the lower risk of eczema are needed.

Supporting information

S1 Table. Food items with a loading score ≥ |0.30| that characterize each of the three dietary patterns. Reprinted with permission[39].
(PDF)

S2 Table. Harmonized definitions. The exact questions as presented on questionnaire completed by mother for each of the cohorts, and a harmonized definition for each covariate.
(PDF)

Acknowledgments

Members of the Nutrigen Alliance: Sonia S. Anand; Stephanie A. Atkinson; Meghan B. Azad; Allan B. Becker; Jeffrey Brook; Judah Denburg; Dipika Desai; Russell J. de Souza; Milan Gupta; Michael Kobor; Diana L. Lefebvre; Wendy Lou; Piushkumar J. Mandhane; Sarah McDonald; Andrew Mente; David Meyre; Theo J. Moraes; Katherine Morrison; Guillaume Paré; Malcolm R. Sears; Padmaja Subbarao; Koon K. Teo; Stuart E. Turvey; Julie Wilson; Salim Yusuf; Stephanie Atkinson; Gita Wahi; Michael A. Zulyniak.

Author Contributions

Conceptualization: Michael A. Zulyniak, Russell J. de Souza.

Data curation: Russell J. de Souza, Keith Tam, Natalie William, Dipika Desai, Diana L. Lefebvre, Malcolm R. Sears.

Formal analysis: Michael A. Zulyniak, Russell J. de Souza, Milan Gupta, Padmaja Subbarao.

Funding acquisition: Michael A. Zulyniak, Russell J. de Souza, Dipika Desai, Diana L. Lefebvre, Padmaja Subbarao, Allan B. Becker, Piushkumar J. Mandhane, Stuart E. Turvey, Koon K. Teo, Malcolm R. Sears.

Investigation: Michael A. Zulyniak, Russell J. de Souza, Dipika Desai, Malcolm R. Sears.

Methodology: Michael A. Zulyniak, Russell J. de Souza, Mateen Shaikh, Chinthanie Ramasundarahettige, Malcolm R. Sears.

Project administration: Russell J. de Souza, Natalie William, Dipika Desai, Malcolm R. Sears.

Resources: Malcolm R. Sears.

Supervision: Russell J. de Souza.

Validation: Michael A. Zulyniak.

Visualization: Michael A. Zulyniak, Russell J. de Souza, Mateen Shaikh.

Writing – original draft: Michael A. Zulyniak.

Writing – review & editing: Michael A. Zulyniak, Russell J. de Souza, Milan Gupta, Padmaja Subbarao, Allan B. Becker, Piushkumar J. Mandhane, Stuart E. Turvey, Theo Moraes, Meghan B. Azad, Koon K. Teo, Malcolm R. Sears.

References

1. Stone KD. Atopic diseases of childhood. Current Opinion in Pediatrics. 2003; 15(5):495–511. https://doi.org/10.1097/00008480-200310000-00009 PMID: 14508299
2. Garner R, Kohen D. Changes in the prevalence of asthma among Canadian children. Health Reports. 2008; 19(2):45. PMID: 18642518
3. Strachan DP. Family size, infection and atopy: the first decade of the "hygiene hypothesis". Thorax. 2000; 55 Suppl 1(Suppl 1):S2–10.
4. Chu LM, Rennie DC, Cockcroft DW, Pahwa P, Dosman J, Hagel L, et al. Prevalence and determinants of atopy and allergic diseases among school-age children in rural Saskatchewan, Canada. Annals of Allergy, Asthma & Immunology. 2014; 113(4):430–9.
5. Dell SD, Foty RG, Gilbert NL, Jerrett M, To T, Walter SD, et al. Asthma and allergic disease prevalence in a diverse sample of Toronto school children: Results from the Toronto Child Health Evaluation Questionnaire (T-CHEQ) Study. Canadian respiratory journal. 2010; 17(1):e1–e6. https://doi.org/10.1155/2010/913123 PMID: 20186360
6. Elbert NJ, Duijts L, den Dekker HT, Jaddoe VW, Sonnenschein-van der Voort AM, de Jongste JC, et al. Role of environmental exposures and filaggrin mutations on associations of ethnic origin with risk of childhood eczema. The Generation R Study. Pediatr Allergy Immunol. 2016; 27(6):627–35. https://doi.org/10.1111/pai.12579 PMID: 27091498
7. Petherick ES, Pearce N, Sunyer J, Wright J. Ethnic and socio-economic differences in the prevalence of wheeze, severe wheeze, asthma, eczema and medication usage at 4 years of age: Findings from the Born in Bradford birth cohort. Respiratory medicine. 2016; 119:122–9. https://doi.org/10.1016/j.rmed.2016.08.017 PMID: 27692132
8. Kull I, Wickman M, Lilja G, Nordvall SL, Pershagen G. Breast feeding and allergic diseases in infants-a prospective birth cohort study. Archives of disease in childhood. 2002; 87(6):478–81. https://doi.org/10.1136/adc.87.6.478 PMID: 12456543
9. Nafstad P, Magnus P, Gaarder PI, Jaakkola JJ. Exposure to pets and atopy-related diseases in the first 4 years of life. 30th Congress of the European Academy of Allergy and Clinical Immunology Istanbul Turkey Conference Start: 20110611 Conference End: 20110615,(varpagings). 2001; 56(4):307–12.
10. Garcia-Larsen V, Ierodiakonou D, Jarrold K, Cunha S, Chivinge J, Robinson Z, et al. Diet during pregnancy and infancy and risk of allergic or autoimmune disease: A systematic review and meta-analysis. PLoS medicine. 2018; 15(2):e1002507. https://doi.org/10.1371/journal.pmed.1002507 PMID: 29489823
11. Beckhaus AA, Garcia Marcos L, Forno E, Pacheco Gonzalez RM, Celedón JC, Castro-Rodríguez JA. Maternal nutrition during pregnancy and risk of asthma, wheeze, and atopic diseases during childhood: a systematic review and meta-analysis. Allergy. 2015; 70(12):1588–604. https://doi.org/10.1111/all.12729 PMID: 26296633
12. Sausenthaler S, Koletzko S, Schaaf B, Lehmann I, Borte M, Herbarth O, et al. Maternal diet during pregnancy in relation to eczema and allergic sensitization in the offspring at 2 y of age. Am J Clin Nutr. 2007; 85(2):530–7. https://doi.org/10.1093/ajcn/85.2.530 PMID: 17284754
13. Miyake Y, Sasaki S, Tanaka K, Ohfuji S, Hirota Y. Maternal fat consumption during pregnancy and risk of wheeze and eczema in Japanese infants aged 16–24 months: the Osaka Maternal and Child Health Study. Thorax. 2009.
14. Garcia-Larsen V, Ierodiakonou D, Jarrold K, Cunha S, Chivinge J, Robinson Z, et al. Diet during pregnancy and infancy and risk of allergic or autoimmune disease: A systematic review and meta-analysis. PLoS medicine. 2018; 15(2):e1002507. https://doi.org/10.1371/journal.pmed.1002507 PMID: 29489823
15. de Souza RJ, Zulyniak MA, Desai D, Shaikh MR, Campbell NC, Lefebvre DL, et al. Harmonization of Food-Frequency Questionnaires and Dietary Pattern Analysis in 4 Ethnically Diverse Birth Cohorts. The Journal of nutrition. 2016; 146(11):2343–50. https://doi.org/10.3945/jn.116.236729 PMID: 27708121

16. Nagao A, Kotake-Nara E, Hase M. Effects of fats and oils on the bioaccessibility of carotenoids and vitamin E in vegetables. Bioscience, biotechnology, and biochemistry. 2013; 77(5):1055–60. https://doi.org/10.1271/bbb.130025 PMID: 23649270
17. Loo EXL, Ong L, Goh A, Chia AR, Teoh OH, Colega MT, et al. Effect of Maternal Dietary Patterns during Pregnancy on Self-Reported Allergic Diseases in the First 3 Years of Life: Results from the GUSTO Study. Int Arch Allergy Immunol. 2017; 173(2):105–13. https://doi.org/10.1159/000475497 PMID: 28654921
18. Hu FB. Dietary pattern analysis: a new direction in nutritional epidemiology. Current opinion in lipidology. 2002; 13(1):3–9. https://doi.org/10.1097/00041433-200202000-00002 PMID: 11790957
19. Quatromoni PA, Copenhafer DL, Demissie S, D'Agostino RB, O'Horo CE, Nam BH, et al. The internal validity of a dietary pattern analysis. The Framingham Nutrition Studies. Journal of epidemiology and community health. 2002; 56(5):381–8. https://doi.org/10.1136/jech.56.5.381 PMID: 11964437
20. Subbarao P, Anand SS, Becker AB, Befus AD, Brauer M, Brook JR, et al. The Canadian Healthy Infant Longitudinal Development (CHILD) Study: examining developmental origins of allergy and asthma. Thorax. 2015; 70:998–1000. https://doi.org/10.1136/thoraxjnl-2015-207246 PMID: 26069286
21. Morrison KM, Atkinson SA, Yusuf S, Bourgeois J, McDonald S, McQueen MJ, et al. The Family Atherosclerosis Monitoring In earLY life (FAMILY) study: rationale, design, and baseline data of a study examining the early determinants of atherosclerosis. American heart journal. 2009; 158(4):533–9. https://doi.org/10.1016/j.ahj.2009.07.005 PMID: 19781411
22. Anand SS, Vasudevan A, Gupta M, Morrison K, Kurpad A, Teo KK, et al. Rationale and design of south Asian birth cohort (START): a Canada-India collaborative study. BMC public health. 2013; 13(1):79.
23. Wahi G, Wilson J, Miller R, Anglin R, McDonald S, Morrison KM, et al. Aboriginal birth cohort (ABC): a prospective cohort study of early life determinants of adiposity and associated risk factors among Aboriginal people in Canada. BMC public health. 2013; 13(1):608.
24. Lankinen MA, Stančáková A, Uusitupa M, Ågren J, Pihlajamäki J, Kuusisto J, et al. Plasma fatty acids as predictors of glycaemia and type 2 diabetes. Diabetologia. 2015; 58(11):2533–44. https://doi.org/10.1007/s00125-015-3730-5 PMID: 26277381
25. Anand SS, Yusuf S, Vuksan V, Devanesen S, Teo KK, Montague PA, et al. Differences in risk factors, atherosclerosis, and cardiovascular disease between ethnic groups in Canada: the Study of Health Assessment and Risk in Ethnic groups (SHARE). The Lancet. 2000; 356(9226):279–84.
26. Fung TT, Rimm EB, Spiegelman D, Rifai N, Tofler GH, Willett WC, et al. Association between dietary patterns and plasma biomarkers of obesity and cardiovascular disease risk. Am J Clin Nutr. 2001; 73 (1):61–7. https://doi.org/10.1093/ajcn/73.1.61 PMID: 11124751
27. Gadgil MD, Anderson CA, Kandula NR, Kanaya AM. Dietary patterns in Asian Indians in the United States: an analysis of the metabolic syndrome and atherosclerosis in South Asians Living in America study. Journal of the Academy of Nutrition and Dietetics. 2014; 114(2):238–43. https://doi.org/10.1016/j.jand.2013.09.021 PMID: 24295929
28. Nettleton JA, Polak JF, Tracy R, Burke GL, Jacobs DR. Dietary patterns and incident cardiovascular disease in the Multi-Ethnic Study of Atherosclerosis. Am J Clin Nutr. 2009; 90(3):647–54. https://doi.org/10.3945/ajcn.2009.27597 PMID: 19625679
29. Iqbal R, Anand S, Ounpuu S, Islam S, Zhang X, Rangarajan S, et al. Dietary patterns and the risk of acute myocardial infarction in 52 countries: results of the INTERHEART study. Circulation. 2008; 118 (19):1929–37. https://doi.org/10.1161/CIRCULATIONAHA.107.738716 PMID: 18936332
30. Huijbregts P, Feskens E, Räsänen L, Fidanza F, Nissinen A, Menotti A, et al. Dietary pattern and 20 year mortality in elderly men in Finland, Italy, and The Netherlands: longitudinal cohort study. Bmj. 1997; 315(7099):13–7. https://doi.org/10.1136/bmj.315.7099.13 PMID: 9233319
31. Kant AK. Indexes of overall diet quality: a review. Journal of the American Dietetic Association. 1996; 96 (8):785–91. https://doi.org/10.1016/S0002-8223(96)00217-9 PMID: 8683010
32. Millen BE, Quatromoni PA, Gagnon DR, Cupples LA, Franz MM, D'Agostino RB. Dietary patterns of men and women suggest targets for health promotion: the Framingham Nutrition Studies. American Journal of Health Promotion. 1996; 11(1):42–51. https://doi.org/10.4278/0890-1171-11.1.42 PMID: 10163450
33. Hu FB, Rimm EB, Stampfer MJ, Ascherio A, Spiegelman D, Willett WC. Prospective study of major dietary patterns and risk of coronary heart disease in men. Am J Clin Nutr. 2000; 72(4):912–21. https://doi.org/10.1093/ajcn/72.4.912 PMID: 11010931
34. Kim J-O, Mueller CW. Factor analysis: Statistical methods and practical issues: Sage; 1978.
35. Northstone K, Ness AR, Emmett PM, Rogers IS. Adjusting for energy intake in dietary pattern investigations using principal components analysis. European journal of clinical nutrition. 2007; 62(7):931–8. https://doi.org/10.1038/sj.ejcn.1602789 PMID: 17522611

36. Willett WC, Stampfer M. Implications of total energy intake for epidemiologic analyses. Nutritional epidemiology1998.
37. IADPSG Consensus Panel. International association of diabetes and pregnancy study groups recommendations on the diagnosis and classification of hyperglycemia in pregnancy. Diabetes care. 2010; 33 (3):676–82. https://doi.org/10.2337/dc09-1848 PMID: 20190296
38. Azur MJ, Stuart EA, Frangakis C, Leaf PJ. Multiple imputation by chained equations: what is it and how does it work? International journal of methods in psychiatric research. 2011; 20(1):40–9. https://doi.org/10.1002/mpr.329 PMID: 21499542
39. Honaker J. Amelia II: A program for missing data. Journal of Statistical Software. 2011; 45(7):1–47.
40. Calov M, Alinaghi F, Hamann CR, Silverberg J, Egeberg A, Thyssen JP. The Association Between Season of Birth and Atopic Dermatitis in the Northern Hemisphere: A Systematic Review and Meta-Analysis. The Journal of Allergy and Clinical Immunology: In Practice. 2019.
41. Zulyniak MA, de Souza RJ, Shaikh M, Desai D, Lefebvre DL, Gupta M, et al. Does the impact of a plant-based diet during pregnancy on birth weight differ by ethnicity? A dietary pattern analysis from a prospective Canadian birth cohort alliance. BMJ open. 2017; 7(11):e017753. https://doi.org/10.1136/bmjopen-2017-017753 PMID: 29138203
42. Miyake Y, Sasaki S, Tanaka K, Hirota Y. Dairy food, calcium and vitamin D intake in pregnancy, and wheeze and eczema in infants. The European respiratory journal. 2010; 35(6):1228–34. https://doi.org/10.1183/09031936.00100609 PMID: 19840962
43. Miyake Y, Okubo H, Sasaki S, Tanaka K, Hirota Y. Maternal dietary patterns during pregnancy and risk of wheeze and eczema in Japanese infants aged 16–24 months: the Osaka Maternal and Child Health Study. Pediatric allergy and immunology: official publication of the European Society of Pediatric Allergy and Immunology. 2011; 22(7):734–41.
44. Chatzi L, Garcia R, Roumeliotaki T, Basterrechea M, Begiristain H, Iñiguez C, et al. Mediterranean diet adherence during pregnancy and risk of wheeze and eczema in the first year of life: INMA (Spain) and RHEA (Greece) mother–child cohort studies. British Journal of Nutrition. 2013; 110(11):2058–68. https://doi.org/10.1017/S0007114513001426 PMID: 23680284
45. Saito K, Yokoyama T, Miyake Y, Sasaki S, Tanaka K, Ohya Y, et al. Maternal meat and fat consumption during pregnancy and suspected atopic eczema in Japanese infants aged 3–4 months: the Osaka Maternal and Child Health Study. Pediatric allergy and immunology: official publication of the European Society of Pediatric Allergy and Immunology. 2010; 21(1 Pt 1):38–46.
46. Lesser IA, Gasevic D, Lear SA. The Association between Acculturation and Dietary Patterns of South Asian Immigrants. PloS one. 2014; 9(2):e88495. https://doi.org/10.1371/journal.pone.0088495 PMID: 24558396
47. Berkowitz AC, J.I S. Atopic Dermatitis. Pediatric Skin of Color. Springer, New York, USA: SpringerLink; 2015. p. 267–80.
48. Zhao CY, Hao EY, Oh DD, Daniel BS, Martin LK, Su JC, et al. Dermatological evaluation in patients with skin of colour: the effect of erythema on outcome measures in atopic dermatitis. British Journal of Dermatology. 2017; 176(4):985–92. PMID: 28012183
49. El-Heis S, Crozier SR, Robinson SM, Harvey NC, Cooper C, Inskip HM, et al. Higher maternal serum concentrations of nicotinamide and related metabolites in late pregnancy are associated with a lower risk of offspring atopic eczema at age 12 months. Clin Exp Allergy. 2016; 46(10):1337–43. https://doi.org/10.1111/cea.12782 PMID: 27517618
50. Würtz P, Wang Q, Kangas AJ, Richmond RC, Skarp J, Tiainen M, et al. Metabolic Signatures of Adiposity in Young Adults: Mendelian Randomization Analysis and Effects of Weight Change. PLoS medicine. 2014; 11(12):e1001765. https://doi.org/10.1371/journal.pmed.1001765 PMID: 25490400
51. Deng Q, Lu C, Li Y, Sundell J, Norbäck D. Exposure to outdoor air pollution during trimesters of pregnancy and childhood asthma, allergic rhinitis, and eczema. Environmental research. 2016; 150:119–27. https://doi.org/10.1016/j.envres.2016.05.050 PMID: 27281689
52. Hart C, Raynor H, Jelalian E, Drotar D. The association of maternal food intake and infants' and toddlers' food intake. Child: care, health and development. 2010; 36(3):396–403.
